# Neurological adverse events of mitotane in adrenocortical carcinoma: results of a pilot study

**DOI:** 10.3389/fonc.2023.1222002

**Published:** 2023-08-14

**Authors:** Marilda Mormando, Edvina Galiè, Marta Bianchini, Rosa Lauretta, Giulia Puliani, Antonio Tanzilli, Umberto Anceschi, Giuseppe Simone, Gianluca Petreri, Giuliana Graziano, Andrea Pace, Marialuisa Appetecchia

**Affiliations:** ^1^ Oncological Endocrinology Unit, IRCCS Regina Elena National Cancer Institute, Rome, Italy; ^2^ Neuro-oncology Unit, IRCCS Regina Elena National Cancer Institute, Rome, Italy; ^3^ Department of Urology, IRCCS, Regina Elena National Cancer Institute, Rome, Italy

**Keywords:** mitotane, adrenocortical carcinoma, neurological toxicity, electroencephalogram, P300, neuropsychological testing

## Abstract

**Introduction:**

Mitotane, the only drug approved by the Food and Drug Administration (FDA) for the treatment of adrenocortical carcinoma, is associated with several side effects including neurotoxicity. The aim of our study is to investigate the relationship between mitotane plasma levels and neurological toxicity.

**Methods:**

We have considered five patients affected by adrenocortical carcinoma treated with mitotane. The neurological assessment included a neurological examination, an electroencephalogram, event-related potentials (P300), and a neuropsychological assessment. All of the patients were first considered at the onset of symptoms of neurotoxicity or when mitotanemia levels were above 18 mg/L, for the second time at mitotanemia normalization and subsequently at its further increase, or in case of persistent neurological abnormalities, some months after normalization.

**Results:**

At the first neurotoxicity, four patients showed impaired neurological examination, electroencephalogram, and P300; three patients had impaired neuropsychological assessment; one patient, only P300. At mitotanemia normalization, the neurological examination became normal in all patients and electroencephalogram normalized in one patient, improved in another one, continuing to be altered in the other three. P300 latency and neuropsychological assessment normalized in two patients and persisted altered in the patient experiencing long-term mitotane toxicity. At the third evaluation, in the patient with prolonged mitotane toxicity, the normal mitotanemia in the previous 9 months restored P300 and improved the electroencephalogram but not the neuropsychological assessment. In the two patients experiencing a further rise of mitotanemia, neurological examination was normal but P300 and electroencephalogram were altered.

**Conclusion:**

The results of our study highlighted the presence of neurophysiological and neuropsychological abnormalities associated with mitotane values above 18 mg/L.

## Introduction

Adrenocortical carcinoma (ACC) is a rare form of endocrine neoplasia with an estimated incidence of 1/1.5–2 million individuals per year. The prognosis of ACC is generally poor especially in the advanced stages, with a 5-year survival <15% for metastatic disease (1). Mitotane, a dichloro-diphenyl-trichloro-ethane derivative, is the only antineoplastic agent for ACC approved by the US Food and Drug Administration and is mainly employed as adjuvant treatment after incomplete surgical resection and for the treatment of advanced ACC (2). Mitotane blood levels must be periodically measured (every 2–4 weeks), and the drug dosage must be titrated in order to reach as quickly as possible a plasma concentration above 14 mg/L because, in various studies, this cutoff has been associated with a prolongation of relapse-free survival (RFS) (3–5). Patients with plasma levels above 20 mg/L have a significantly increased risk of experiencing severe side effects; therefore, the therapeutic window between 14 and 20 mg/L has been proposed and approved (6). The daily dose usually ranges between 2 and 10 g (7). The most common side effects of mitotane therapy are gastrointestinal, endocrine, and neurological. Mitotane, in high doses, can cause neuromuscular symptoms manifesting as ataxia, speech disturbances, confusion, somnolence, depression, memory deficits, muscle tremors, polyneuropathy, and dizziness (8). Although several studies and drug vigilance data have demonstrated and described a neurological toxicity due to mitotane (9), there are only anecdotal cases of neurological toxicity documented by instrumental examinations such as brain magnetic resonance imaging (MRI) or electroencephalogram (EEG). Furthermore, from the literature data, both the level of mitotanemia to which the appearance of neurological symptoms is most frequently associated and a more precise characterization of the same remain unclear due to the lack of instrumental data and neuropsychological tests in patients who have developed neurological toxicity related to mitotane therapy.

## Subjects and methods

A retrospective/prospective monocentric study has been conducted in patients affected by ACC treated with mitotane. Since it is a rare disease, 10 patients affected by ACC are followed up in our department: three of them have no indication for mitotane therapy while the other seven take mitotane for adjuvant purposes or for metastatic disease. Among these seven patients, two were excluded from the study, since they were recently enrolled and have not yet reached therapeutic levels of mitotanemia nor have they developed neurological symptoms from drug toxicity. In this study, we have analyzed the preliminary retrospective results of the first five patients enrolled.

The inclusion criteria for this study were as follows:
- Patients diagnosed with ACC receiving mitotane therapy both for adjuvant purposes and in the presence of metastatic disease, associated or not with other anticancer treatments
- Patients with suspected symptoms of neurological toxicity and/or detection of mitotanemia levels in the high limits or above the therapeutic range (>18 mg/L)
- Patients signed an informed consent form
The exclusion criteria for this study were as follows:
- Central nervous system disorders of other nature
- Mitotanemia <18 mg/L with absence of neurological signs/symptoms

All of the five patients have been considered for the first time at the onset of signs/symptoms of neurotoxicity and/or when mitotane serum concentration was >18 mg/L. The neurological assessment included a neurological examination (NE), an EEG, event-related potentials (P300), and a neuropsychological assessment (NA).

They have been tested a second time just at the restoring of normal mitotane plasma levels and subsequently at the time of a further increase in mitotanemia or, in case of persistent neurological abnormalities, some months after mitotanemia normalization.

Mitotane concentrations were measured by Lysosafe^®^ service, a free-of-charge service offered by HRA Pharma to European prescribers and associated with the use of Lysodren^®^. Samples were collected in our center, sent to a centralized laboratory, extracted by precipitation with ethanol, and tested by a standardized gas chromatography/mass spectrometry method.

Mitotanemia blood levels have been measured routinely, in particular after approximately 30 days from the start of therapy with mitotane, and periodically, every 30–60 days and/or in any case according to clinical judgment. At the time of each mitotanemia assessment, clinical examination and blood laboratory tests including thyroid and adrenal function, liver and kidney function, and electrolytes were performed. These hematic tests were essential to discriminate against the nature of neurological symptoms, if present.

### Neurological assessment

#### Electroencephalogram

EEG is one of the standard methods to measure electrical brain activity in many fields and the main source of signals for noninvasive brain–computer interfaces.

In our laboratory, we use electrodes that get mounted at the scalp level with the help of an EEG cap, located in the standard position 10-20 (10, 11). EEG recordings lasted 30 min that included hyperventilation and intermittent photic stimulation.

#### Event-related potential (P300)

P300 is a cognitive potential belonging to long latency auditory evoked potentials. It corresponds to the largest positive wave after the N1-P2 complex registered by surface electrodes placed at the scalp level with international 10-20 system position. The P300 component is elicited by the recognition of a rare random deviant auditory stimulus (oddball paradigm) within a series of frequent similar auditory stimuli at 80 ± 10 Decibel Sound Pressure level (dB) that should be mentally counted. It can be used to identify the functional integrity, the level of impairment, or the degree of maturation of visual, auditory, somatosensory peripheral nerve pathways and of higher cognitive functions. Normal latency values of our laboratory are from 305 to 320 ms with no difference between the left and right ears (12, 13).

### Neuropsychological assessment

Neurocognitive functions were assessed by a battery of standardized tests lasting approximately 60 min in order to balance brevity and sensitivity (14). Tests used were the Digit and Corsi Span Test (forward and backward) (15), Rey Auditory Verbal Learning Test-Recall and logical memory, immediate and delayed recall (16), Rey-Osterrieth Complex Figure copy and recall (17), Raven’s Colored Progressive Matrices 38 (CPM) (18), semantic and phonemic fluency (19), and Trail-Making test, part A and part B (20).

For cognitive measures, age-, gender-, and education-corrected scores and equivalent scores were calculated from the raw scores according to Italian normative standards. In order to accomplish data reduction for statistical purposes and following a hierarchical approach, individual test scores were grouped into seven cognitive domains: verbal learning (VL), short-term memory (STM), long-term memory (LTM), executive functions (EFs), abstract reasoning (AR), attention (A), and visuo-constructional abilities (VCAs).

Participants were coded as “non-impaired” or “impaired” in each single domain according to a pathological equivalent score corresponding to 1,65 standard deviations below the average score of normative populations.

## Results

### Patient 1

**Figure 1:**
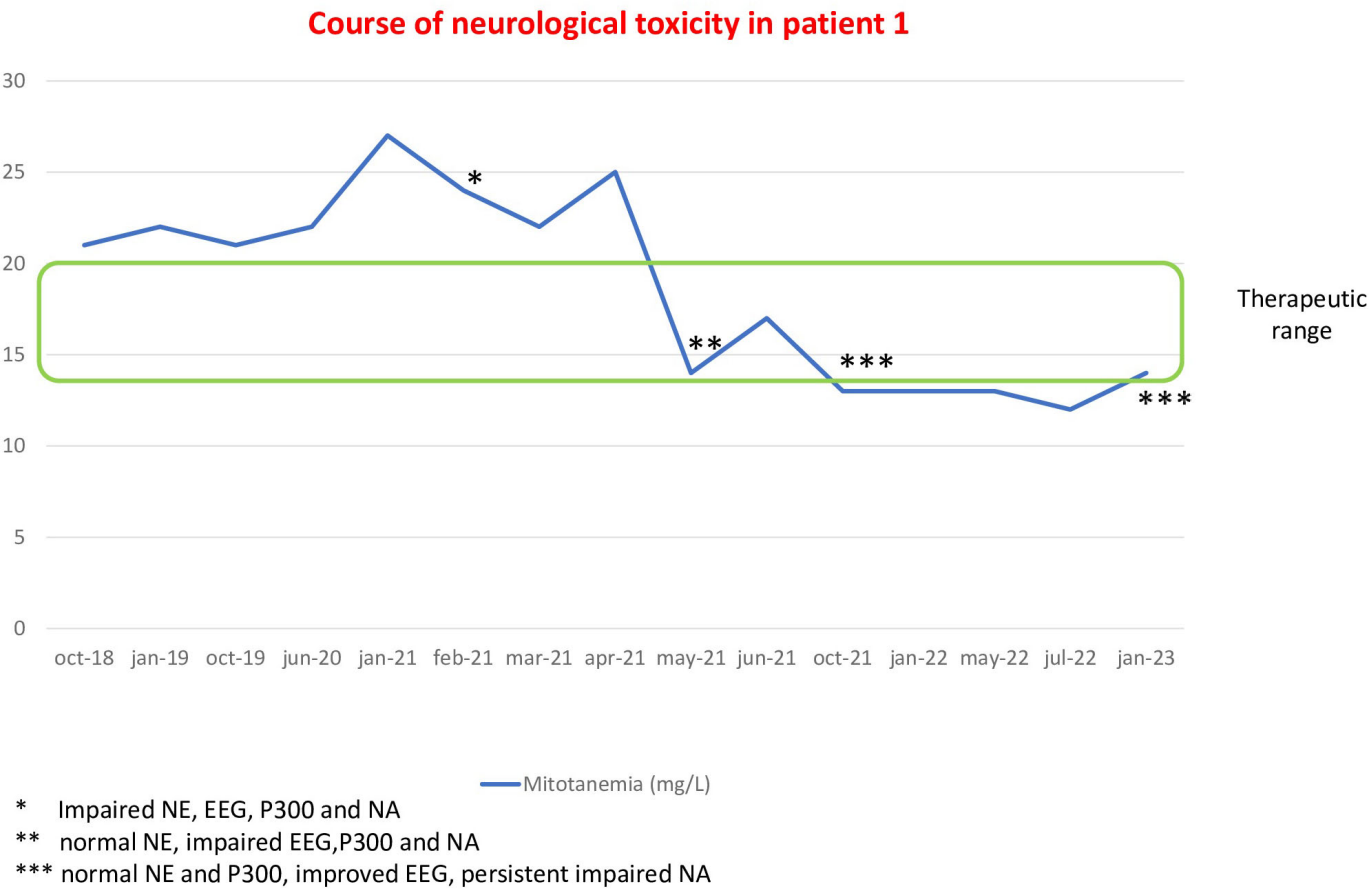
Course of neurological alterations associated with mitotane blood levels in patient 1.

Patient 1 is a 36-year-old woman who started mitotane therapy in August 2014 at the age of 29 after adrenalectomy for ACC. In 2016, she underwent stereotaxic radiation treatment for a lung metastatic lesion without discontinuation of mitotane. At the first neurological evaluation, in February 2021, mitotane blood levels were above the therapeutic threshold (23.9 mg/L) for more than 2 years at dose of 3 g daily. The NE showed an ideomotor slowdown and a mild short-term memory deficit. At the EEG, epileptic abnormalities were found, and the P300 exam showed an increased latency. At the NA, the patient showed a selective visual-constructive deficit and visual memory deficit. At the time of neurological toxicity, thyroid and adrenal function tests were normal, well compensated by replacement therapy with levothyroxine and hydrocortisone. An MRI was performed to exclude metastatic cerebral lesions. After 3 months, at the normalization of mitotane levels (17.4 mg/L) with a reduced dose of 1.5 g, the NE was normal and the NA slightly improved, with persistence of the same visuo-constructive deficit. The EEG and P300 assessment, on the other hand, were confirmed impaired. Only 9 months after mitotanemia normalization, the patient experienced a normal P300 and an improvement of EEG, even though slight bioelectric abnormalities persisted in occipito-temporo-parieto regions. Also, the selective visual-constructive deficit was confirmed at NA. At the last neurological assessment, maintaining mitotane levels within the therapeutic window for 20 months, in January 2023, the NE and P300 were normal, slight abnormalities were confirmed at the EEG as well as the visual-constructive deficit at NA, even milder than at the time of toxicity (**Figure 1**).

### Patient 2

**Figure 2:**
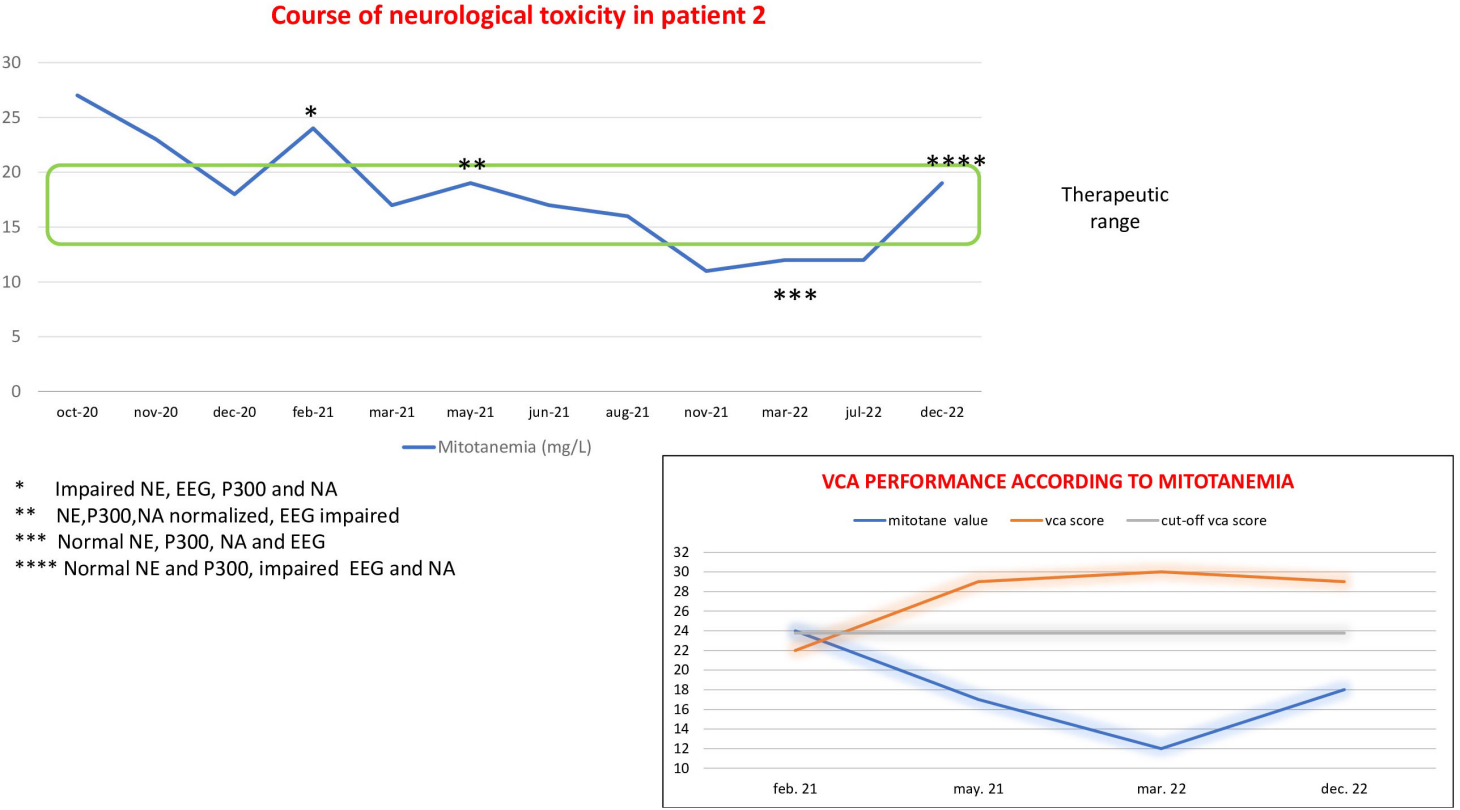
Course of neurological alterations associated with mitotane blood levels and trend of visuo-constructional abilities (VCAs) according to mitotanemia in patient 2.

Patient 2 is a 60-year-old woman who underwent a left adrenalectomy in 2017 for ACC and a resection of a liver metastasis in 2018. In 2019, a metastatic lesion in the iliac bone was treated with embolization and local radiotherapy. Mitotane treatment was started in 2018 and discontinued in 2019, then resumed in July 2020. In October 2020, mitotane levels were over the therapeutic threshold (26.8 mg/L) with a daily dose of 4 g, but neurological assessment was performed only after 4 months, in February 2021, when mitotane concentration was 24 mg/L at the dose of 2.5 g. The first NE showed a general slight slowdown, and the EEG was indicative of a slight slowing of the background activity with anomalies on the temporal-parieto-occipital regions. The latency of the P300 exam was at the upper limits of normal, and the NA showed a selective visual-constructive deficit. Thyroid and adrenal function tests were normal, well compensated by replacement therapy. Two months later, at the dose reduction to 1.5 g/day, mitotane levels dropped to normal range (17 mg/L); then, the NE, P300, and NA normalized, but the EEG showed only slight nonspecific anomalies without slowing down of the background activity. In March 2022, when mitotanemia dropped below the normal limit (12 mg/L), neurological assessment was completely normal, including EEG. At the latest neurological evaluation, in December 2022 (mitotanemia 18 mg/L), the NE and P300 were normal, whereas the EEG showed a slight slowdown of background activity with nonspecific anomalies in the temporo-parieto-occipital regions and the NA demonstrated a short-term verbal memory deficit (**Figure 2**).

### Patient 3

**Figure 3:**
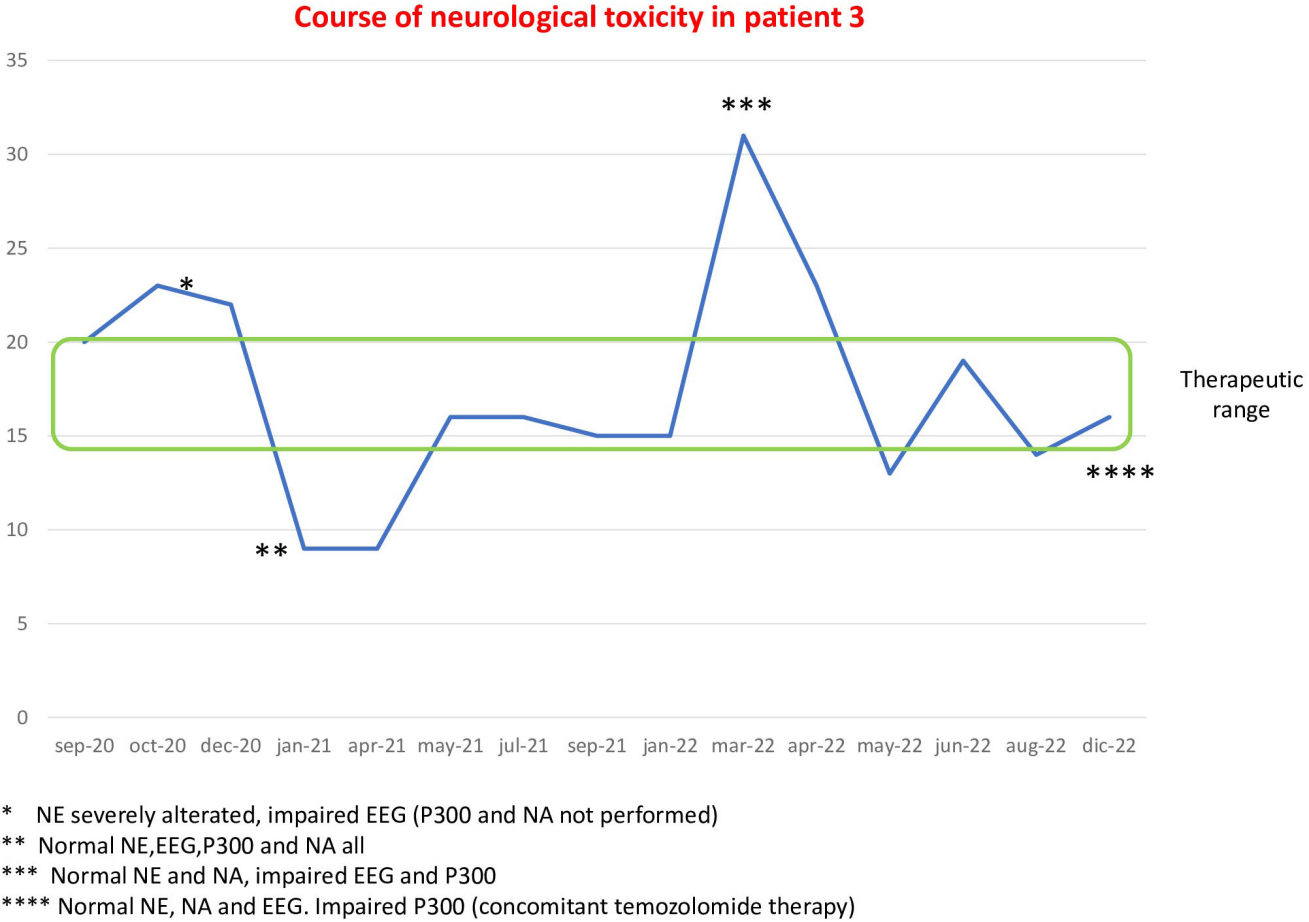
Course of neurological alterations associated with mitotane blood levels in patient 3.

Patient 3 is a 50-year-old man who, in December 2019, underwent surgical removal of a 4-cm retroperitoneal mass diagnosed as ACC. Due to the presence of lung metastases at diagnosis, the patient was immediately initiated mitotane treatment (in April 2020) combined with cisplatin and etoposide chemotherapy (started in May 2020). After 8 months of mitotane therapy at a dose until 4.5 g, in November 2020, the patient developed a severe clinical neurological toxicity characterized by spatio-temporal disorientation and memory disturbances. During the NE, the patient presented with a mild disturbance of concentration and bilateral ideomotor apraxia. The EEG showed a diffuse slowing of brain electrical activity with mild diffuse brain distress. Blood levels of mitotanemia were increased (23 mg/L), then a discontinuation of treatment was necessary. A computed tomography scan ruled out the presence of brain metastases. The NA and P300 were not carried out at the time of clinical neurological toxicity. One month after the interruption of mitotane, when mitotanemia levels dropped to 9 mg/L, the NE, EEG, and NA showed no anomalies (P300 not performed). From March to June 2021, the patient underwent a further four cycles of chemotherapy with doxorubicin, cisplatin, and etoposide and, for progression of disease, in March 2022, a second-line systemic treatment with temozolomide, even in combination with mitotane, was started and it is still ongoing. In March 2022, mitotanemia levels increased to 31 mg/L without neurological symptoms. The NE was found normal as well as NA, but EEG showed again a diffuse slowing of electrical activity with mild diffuse distress, and P300 significantly increased. Mitotanemia normalized after 2 months (13 mg/L in May 2022), and a new neurological assessment was performed 7 months after this normalization (December 2022), when temozolomide therapy was ongoing, showing that P300 persistently increased, without anomalies of NE, EEG, and NA (**Figure 3**).

### Patient 4

**Figure 4:**
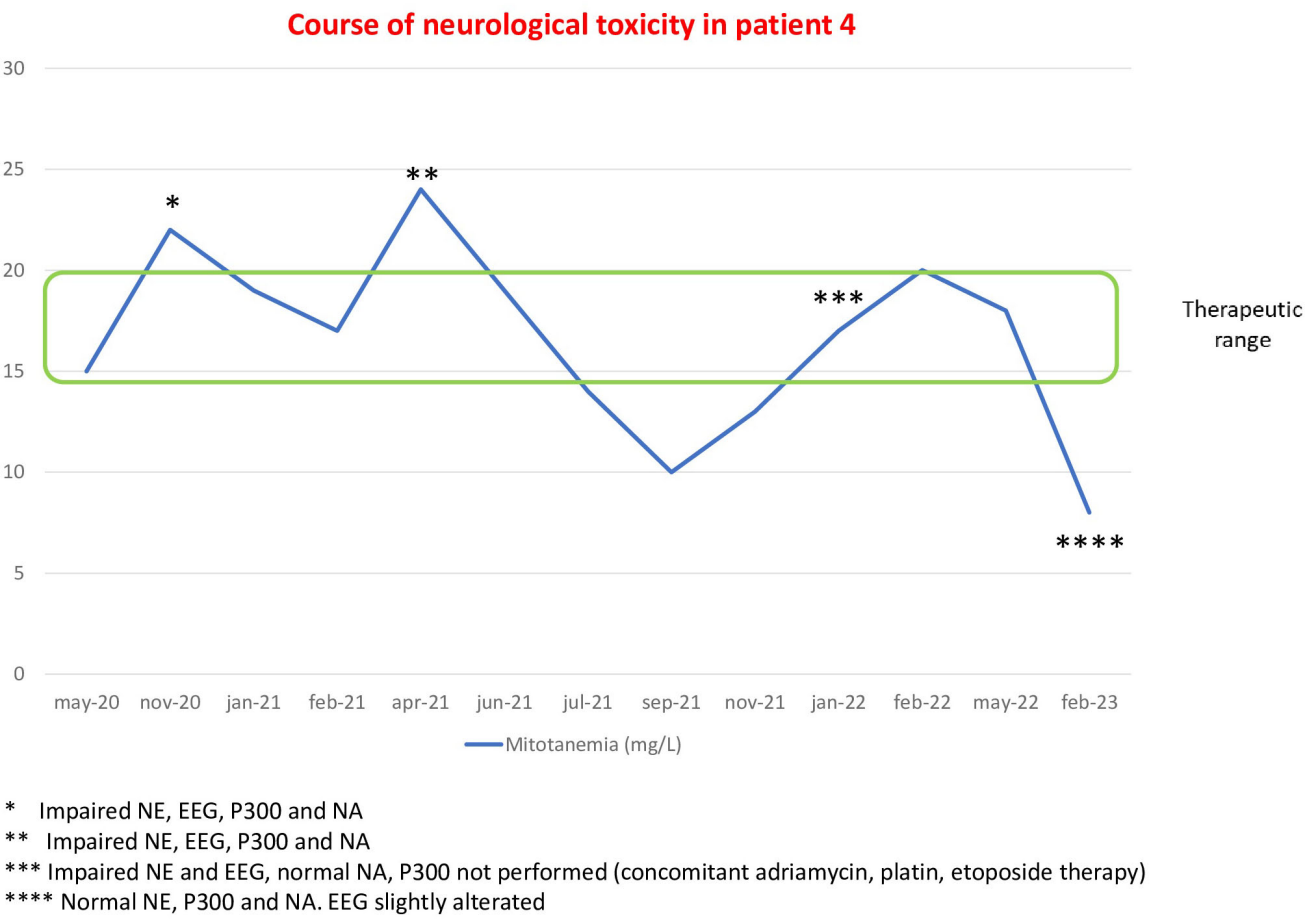
Course of neurological alterations associated with mitotane blood levels in patient 4.

Patient 4 is a 52-year-old woman who underwent left adrenalectomy and ipsilateral nephrectomy with paraaortic lymphadenectomy in November 2019 for ACC. She started adjuvant mitotane from February 2020 and, in November 2020, the drug blood levels were raised to 21.6 mg/L at a mitotane daily dose of 2.5 g. The NE revealed slower thinking, difficulty in concentration, and confusion; the EEG showed slight nonspecific anomalies; P300 was slightly increased; and at NA, a selective visual-constructive deficit and an impairment of visual memory and executive functions were found. In February 2021, she underwent surgical removal of a relapsing lesion in the left adrenal region. She was reassessed in April 2021, at the second cross-threshold mitotanemia finding (24 mg/L), and the same previous abnormalities at NE, EEG, P300, and NA were confirmed. Mitotanemia normalized in June 2021 and remained in the therapeutic range until January 2022, when the patient completed chemotherapy with cisplatin, doxorubicin, and etoposide that started in July 2021 for progression of disease, and neurological assessment was repeated. A collateral neurotoxicity characterized by cognitive and somatosensorial disturbances, associated with ataxia, deficit of attention and concentration, and bilateral hearing loss were evident at NE. Mitotanemia was 16 mg/L, the EEG showed slight nonspecific anomalies, NA was normal, but P300 was not performed. Due to abdominal disease progression, the patient underwent surgical removal in January 2023 of two massive abdominal recurrences of ACC, temporarily discontinuing mitotane therapy from December 2022 to February 2023. In washout of mitotane and chemotherapy, NE was normal as well as P300 and NA, while at EEG, slight nonspecific anomalies in the bilateral fronto-centro-temporal regions persisted (**Figure 4**).

### Patient 5

Patient 5 is a 33-year-old woman, the youngest patient of this series, who in April 2021 underwent left adrenalectomy for a 6-cm ACC lesion. Due to a possible incomplete resection (fragmented neoplasm and therefore not fully assessable), the patient underwent radiotherapy on the tumor bed and on adjacent lymph nodes and started mitotane treatment in an adjuvant setting in April 2021. Drug blood levels did not reach the therapeutic range until February 2022. In March 2022, mitotanemia reached 18.5 mg/L at a dose of 6 g and NA was performed: NE, EEG, and NA were all normal but P300 was increased. The same abnormalities were demonstrated in June 2022 when mitotanemia levels were further raised to 20 mg/L. In December 2022, when drug blood levels decreased to 10 mg/L, also P300 returned to perfectly normal.

## Discussion

The use of mitotane as adjuvant therapy in ACC is still controversial also due to the absence of randomized controlled clinical trials that can support its effectiveness. Since the recurrence rates and the onset of distant metastases are very high, it is often necessary to associate conventional chemotherapy with etoposide, doxorubicin, and cisplatin with mitotane. A recent meta-analysis (21) including five retrospective studies and 1,249 patients demonstrated that adjuvant therapy with mitotane results in a significant prolongation of RFS.

Despite a long-standing clinical use, the molecular mechanisms underlying mitotane efficacy are still poorly understood. Mitotane exerts its action through a selective damage of the corticoadrenal tissue, particularly of the fasciculated and reticular area, less frequently of the glomerular area (22). Recently, it has been demonstrated that mitotane induces apoptosis through stress on the endoplasmic reticulum and inhibition of mitochondrial respiration in cancer cells, by inducing a defect in cytochrome C oxidase activity and inhibiting sterol-O-acyltransferase I (23, 24).

A recent study found that mitotane and its main metabolite bind to lipid membranes by inserting into the lipid–water interface of the bilayer and causing an alteration of the membrane integrity, leading to an increased permeability for polar molecules. This could help to better explain even the side effects of this drug (25).

Mitotane is a lipophilic drug with a low absorption rate and an extremely long half-life (24, 26). These features explain why it takes some weeks or months to reach a steady-state concentration. In clinical tests, the maximum absorption of the drug was observed when associated with a hyperlipidic diet (26), and this is the reason why patients are advised to take mitotane with fatty products such as milk. The 35%–40% of mitotane absorbed from the gastrointestinal tract is distributed within adipose tissue (27). In a study of 19 patients receiving a dose of mitotane of 3 to 6 g/day for 30–60 days, the half-life was shown to be between 18 and 159 days (26) possibly due to its accumulation in adipose tissue and a gradual return to plasma. The polymorphism of CYP enzymes could be involved in the individual variability of the therapeutic effect of mitotane. It has been demonstrated that CYP2B6 is a key cytochrome for mitotane metabolism and that its genetic profile can predict the circulating concentration of mitotane (28–30). In fact, patients with the wild-type “G” allele achieved lower mitotane concentrations than the patient carrying the mutant “T” allele (28).

Mitotane blood concentrations should be maintained between 14 and 20 mg/L because above this (but in some patients also above 15 mg/L), the drug is toxic (24). Mitotane side effects can include the gastrointestinal, endocrine, and nervous system. The gastrointestinal toxicity is characterized by diarrhea, vomiting, nausea, anorexia, mucositis, and increases in hepatic enzyme or bilirubin levels (31). The most common endocrine side effects include hypoadrenalism, hypothyroidism, and hypogonadism/gynecomastia in male patients (32–37).

A significant increase in neurological toxicity has been reported when plasma mitotane levels exceed 20 mg/L. The French National pharmacovigilance database reported from 2004 to 2016 14 cases of neurological adverse events including asthenia, drowsiness, memory disorders, confusion, headaches, and space–time disorientations (9). There are no literature data that allow to define the mitotanemia values beyond which it is necessary to carry out more in-depth neurological investigations nor the duration of mitotane therapy that would increase the risk of neurological toxicity. There are only a few anecdotal cases of neurological toxicity documented by instrumental examinations such as brain MRI/CT scan or EEG and a single dated study in which the neuropsychological aspect was also evaluated.

In 1992, in fact, Bollen and Lanser (38) described accurately for the first time the mental deterioration and neurological disturbances in a 40-year-old woman during mitotane therapy at a daily dose of 2–3 g. During mitotane therapy (drug blood levels measured in two times, respectively, of 32.4 and 18.3 mg/L), the patient developed cerebellar ataxia and showed brisk tendon reflexes. The EEG showed abundant delta waves and beta activity and NA was impaired, proportionally to mitotane excess, the latter resulting ameliorated at a serum mitotane level of 15.6 mg/L and completely restored after drug discontinuation (mitotanemia 6.2 mg/L). Both neurological symptoms and EEG normalized 1–2 months after the end of therapy. The same work group published its complete experience including eight patients, treated for at least 2 years with mitotane at doses leading to drug levels of 20–25 mg/L (39). In all patients, when mitotanemia exceeded 15 mg/L, a neuropsychological impairment especially in visual-spatial tasks, oral language, and memory, a cerebellar ataxia, and EEG abnormalities were found, despite a normal CT brain scan. At mitotane discontinuation, the five patients reevaluated showed a complete recovery of neurological toxicity. In pediatric settings, Goto et al. (40) described in 2008 the case of a 4-year-old boy with ACC treated with a high dose of adjuvant mitotane (up to 5 g/day) who developed difficulty walking and speaking, memory disturbances, and spastic quadriplegia/ataxia. The brain MRI showed mild cerebral and cerebellar atrophy while the EEG diffuse slowing of background activity. At mitotane discontinuation, neurological symptoms disappeared completely 6 months later. A case of neurological toxicity, characterized by attention-deficit hyperactivity disorder, has been reported also in a 5-year-old girl at a low dose of mitotane (1.25 g/m^2^/day) and with drug plasma concentration below the therapeutic range (41). At a mitotanemia increase (24 mg/L), the neurological symptoms worsened, and the patient developed daytime sleepiness, memory disturbances, slurred and repetitive speech, ataxia, and tremors. The EEG showed continuous generalized fast activity, but the pituitary MRI and cerebrospinal fluid analyses revealed no abnormalities. The neurological symptoms improved when the mitotane level decreased below the subtherapeutic threshold.

In our study, a neurological complete assessment was performed in five patients affected by ACC and experienced mitotane toxicity.

Clinically, the most serious neurological signs/symptoms, characterized by space–time disorientation and short-term memory disturbances, were observed in only one patient, disappearing within a few days after discontinuation of mitotane (patient 3). Even patient 4 complained of confusion, dizziness, and instability in walking, but such symptomatology was much more nuanced and fluctuating during the course of therapy. The patient with longer-term mitotane toxicity never complained of neurological symptoms, although at the first evaluation, all of the tests were strongly altered probably due to the chronic excess of mitotanemia (patient 1). Patient 2 and patient 5 did not have any neurological symptoms even with drug blood levels above the threshold. Patient 5 reached the therapeutic window with difficulty and slowly, even with a dose of mitotane up to 6 g daily and at a drug level of 18.5 mg/L, the only impaired test was the P300, immediately reversible after the normalization of mitotanemia.

We have confirmed that neurological toxicity can occur at any dose of mitotane, although a more serious and persistent toxicity is associated with higher doses. Patient 3 developed neurological symptoms with a dose of 4.5 g, while patient 1, in whom the most serious and persistent instrumental alterations were found for a long time, had been treated in the last 4 years with daily doses between 4.5 and 3 g.

We demonstrated that the NE, generally characterized by disturbance of concentration, confusion, slower thinking, ideomotor slowdown, and mild short-term memory deficit, and in worst case by bilateral ideomotor apraxia, was impaired when mitotanemia levels were well beyond the threshold (from 21 to 24 mg/L) and normalized rapidly, as soon as the drug blood levels returned to normal.

The EEG is the most sensitive examination and was found to be altered even for mitotanemia levels at the upper normal limits (18 mg/L). The scale of alteration proved to be proportional to the level of toxicity, since in patient 1, after 7 years of therapy and 2 years of toxicity, the EEG showed even epileptic abnormalities. The EEG alterations were always reversible except in patient 1 in whom the epileptic abnormalities persisted despite the normalization of mitotanemia and only an improvement but not a complete normalization of the EEG was observed even 20 months after the restoration of mitotanemia values in the therapeutic window. In one of the five patients, the EEG was always normal, even in the presence of mitotanemia values over the threshold, and this can be explained by the young age of the patient or related to the difficulty in reaching the therapeutic range of the drug and consequently with a probable low tendency to accumulate in the brain tissues.

Among the neurological instrumental assessments, we evaluated for the first time, in patients with neurological mitotane toxicity, the P300. The P300 test was always altered in all of the five patients at drug levels above 20 mg/L and normalized along with mitotanemia except in patient 1 in whom it was restored only 9 months after the normalization of mitotanemia. The P300 resulted increased despite a mitotanemia in range in patient 3, and we can hypothesize a toxic effect of the concomitant therapy with temozolomide.

Regarding the neuropsychological aspect, the NAs were altered in three out of five patients, at the time of toxicity and for mitotanemia values >18 mg/L, but normalized with mitotanemia values, except for patient 1, also probably influenced by persistently high levels of drugs in the previous years. Interestingly, and in agreement with previous literature data by Bollen and Lanser (38), the neuropsychological deficit involved the visual-constructive domain in all three cases.

At baseline assessment, patients struggled to set up the task, adopting a poorly functional copying strategy (element by element), without an evident execution plan and with poor overall results. Interestingly, copying strategies often improved with normalization of mitotanemia values. Improvement was particularly evident from a qualitative point of view, thus suggesting that performance gain could not be based on practice effect alone. Visual memory, executive (patient 4), and short-term verbal memory deficit (patient 2) occurred less frequently.

The occurrence of neurological side effects has not been associated with electrolyte disturbances, thyroid function alterations, or hypoadrenalism, all of which are adverse events of mitotane therapy. All patients, in fact, despite the occurrence of iatrogenic hypothyroidism and hypoadrenalism, had normal levels of thyroid and adrenal hormones, well compensated by replacement therapy at every time of neurological assessment. None of the patients was taking drugs interfering with mitotane metabolism, especially those metabolized by cytochrome P450 (three patients were taking rosuvastatin or ezetimibe for mitotane-induced hypercholesterolemia).

Although the molecular mechanisms underlying mitotane neurological toxicity are still poorly understood, we can speculate that the lipophilicity of the drug may play a role in facilitating across the blood–brain barrier. Additionally, the central nervous system, rich in lipid, can favor the accumulation of lipophilic molecules such as mitotane, but the amount of this accumulation is not yet currently measurable. Nevertheless, it could be hypothesized that the neurotoxic damage could be due to a direct cytotoxic drug-effect on the myelin sheaths, since it has been demonstrated that mitotane can alter the lipid membrane structure (25).

The major limitation of this study is the low number of patients, which prevented reaching precise conclusions but allowed us to generate hypotheses that provide input to further investigations and studies with a larger number of patients. Unfortunately, not all of the tests were carried out in all of the patients, for logistic reasons, at the time of toxicity and at the time of its resolution. Due to the rarity of the disease, the use of chemotherapeutic agents was not an exclusion criterion, then the neurotoxicity of concurrent chemotherapeutic agents cannot be excluded. We aim to continue this prospective study by enrolling more patients in order to provide additional data regarding the neurological toxicity of mitotane. Mitotane pharmacogenetics could be an additional tool to maintain drug concentrations in the therapeutic range and adjust the right dose to each patient. The detection of the allelic cytochrome CYP2B6 variants could add useful information for both therapy optimization and evaluation of a possible association between them and neurotoxicity; this could be a starting point for further studies.

## Conclusions

In this study, we confirmed that mitotane toxicity is characterized by neurophysiological and neuropsychological abnormalities.

We also demonstrated how the short-term mitotane toxicity results in reversible neurological alterations, while, in cases of years-long toxicity, they can persist for a long time and some of them (as EEG and NA) could be irreversible and significantly affecting the patient’s quality of life.

This study confirmed the importance of frequent monitoring of mitotanemia, which is currently the only way to avoid long periods of overtreatment and to prevent neurological toxicity that could be irreversible. We demonstrated that EEG and NA are very sensitive as indicators of neurological damage, since they can be altered at mitotane blood levels >18 mg/L. For this reason, we should consider a new mitotane therapeutic threshold of 18 mg/L in order to minimize the possibility of neurological toxicity. The discovery of these neurological anomalies, particularly the visuo-constructive deficit, could lead to conducting studies that can better investigate the mechanism of action of this drug, still unknown in some respects, and the possible accumulation in lipidic tissues and in specific brain areas.

## Data availability statement

The datasets presented in this study can be found in online repositories. The names of the repository/repositories and accession number(s) can be found below: GARRbox.

## Ethics statement

The studies involving humans were approved by the Ethics Committee of IRCCS Regina Elena National Cancer Institute. The studies were conducted in accordance with the local legislation and institutional requirements. The participants provided their written informed consent to participate in this study. Written informed consent was obtained from the individual(s) for the publication of any potentially identifiable images or data included in this article.

## Author contributions

MM, EG, and AP contributed to conception and design of the study. MM, EG, MB, RL, GPu, AT, UA, GS, GPe, and GG collected and interpreted the data. MM wrote the first draft of the article. EG and AT wrote sections of the article. MA reviewed and supervised the article. All authors read and approved the final article.
